# Exploratory Studies on Development of the Chemokine Receptor CXCR4 Antagonists Toward Downsizing

**DOI:** 10.4137/pmc.s422

**Published:** 2008-02-10

**Authors:** Hirokazu Tamamura, Hiroshi Tsutsumi, Wataru Nomura, Nobutaka Fujii

**Affiliations:** 1 Institute of Biomaterials and Bioengineering, Tokyo Medical and Dental University, Chiyoda-ku, Tokyo 101-0062, Japan; 2 Graduate School of Pharmaceutical Sciences, Kyoto University, Sakyo-ku, Kyoto 606-8501, Japan

**Keywords:** cancer metastasis, chemokine receptor, CXCR4 antagonist, downsizing, HIV infection, rheumatoid arthritis

## Abstract

Seven transmembrane (7TM) G-protein-coupled receptor (GPCR) families are important targets for drug discovery, and specific antagonists for GPCR can accelerate research in the field of medicinal chemistry. The chemokine receptor CXCR4 is a GPCR that possesses a unique ligand CXCL12/stromal cell-derived factor-1 (SDF-1). The interaction between CXCL12 and CXCR4 is essential for the migration of progenitor cells during embryonic development of the cardiovascular, hemopoietic and central nervous systems, and also involved in several intractable disease processes, including HIV infection, cancer cell metastasis, progression of acute and chronic leukemias, rheumatoid arthritis and pulmonary fibrosis. Thus, CXCR4 may be an important therapeutic target in all of these diseases, and various CXCR4 antagonists have been proposed as potential drugs. Fourteen-mer peptides, T140 and its analogs, and downsized cyclic pentapeptides have been developed by us as potent CXCR4 antagonists. This article describes the development of a number of specific CXCR4 antagonists in our laboratory, including downsizing.

## Introduction

**Figure 1:**
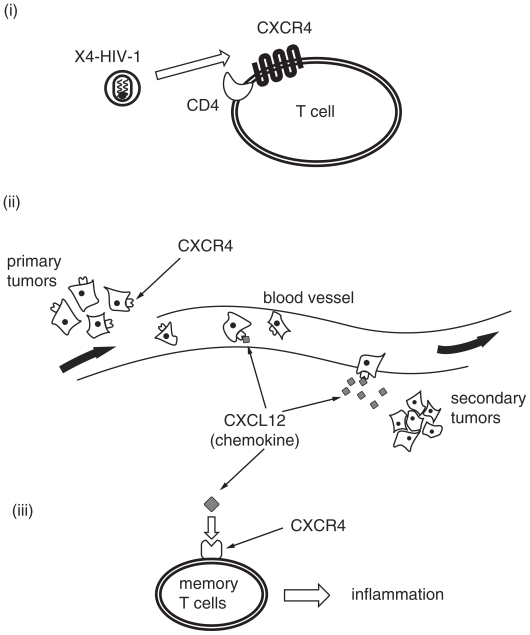
Various disorders relevant to the CXCL12-CXCR4 axis, such as HIV infection (i), cancer cell metastasis (ii) and rheumatoid arthritis (iii).

As a postgenome project, proteomics has been prosperous in life science, and selective ligands involving protein networks have been valuable and useful for studies on chemical biology. Seven transmembrane G-protein-coupled receptors (7TM-GPCRs) are great targets for drug discovery and chemical biology. Thus, development of selective antagonists against each GPCR is extremely desirable (Tamamura and Tsutsumi, 2006). Chemokines are a chemotactic cytokine family that induces migration of leukocytes. Receptors of chemokines, which transduce signals of the corresponding chemokines, are classified into GPCR families. The relationships between chemokines and their receptors are highly interconnected and complicated: in most cases, a single chemokine recognizes a plurality of receptors, and one chemokine receptor recognizes several chemokines. Thus, most of chemokines lack receptor selectivity. However, the chemokine CXCL12/stromal cell-derived factor-1 (SDF-1) possesses the chemokine receptor CXCR4 as its solitary receptor (Tashiro et al. 1993; Nagasawa, Kikutani and Kishimoto, 1994; Oberlin et al. 1996; Bleul et al. 1996). The interaction between CXCL12 and CXCR4 plays a fundamental role in the migration of progenitor cells during embryonic development of the hemopoietic, intestine vascular, cardiovascular and central nervous systems. Its physiological roles in adults remain poorly disclosed. It is also known that the CXCR4-CXCL12 pair is involved in various disease processes such as HIV infection (Feng et al. 1996), cancer cell metastasis (Koshiba et al. 2000; Geminder et al. 2001; Müller et al. 2001; Robledo et al. 2001; Sanz-Rodriguez, Hidalgo and Teixido, 2001; Scotton et al. 2001; Bertolini et al. 2002; Kijima et al. 2002; Schrader et al. 2002; Scotton et al. 2002; Taichman et al. 2002; Burger et al. 2003; Rubin et al. 2003; Tamamura, Hori et al. 2003; Takenaga et al. 2004; Mori et al. 2004; Piovan et al. 2005; Zannettino et al. 2005), progression of acute and chronic leukemias (Tsukada et al. 2002; Juarez et al. 2003; Burger et al. 2005; Spoo et al. 2007) and rheumatoid arthritis (Nanki et al. 2000) (Fig. 1).

CXCR4 was initially identified as a second receptor (co-receptor) of T cell line-tropic (X4-) HIV-1 entry through its association with the first receptor, CD4. Macrophage-tropic (R5-) HIV-1 strains, which use the chemokine receptor CCR5 as another co-receptor, are major in early stages of HIV infection (Alkhatib et al. 1996; Choe et al. 1996; Deng et al. 1996; Doranz et al. 1996; Dragic et al. 1996). However, X4 HIV-1 strains become dominant in the late stages. Recently, it has also been reported that CXCL12 is highly expressed in several internal organs that are the primary targets of cancer cell metastasis, and that CXCR4 is overexpressed on the surfaces of several types of cancer cells. Thus, it has been shown that the CXCL12-CXCR4 axis is associated to metastasis of several types of cancer including cancer of the pancreas, breast, lung, kidney, and prostate and non-Hodgkin’s lymphoma, neuroblastoma, melanoma, ovarian cancer, multiple myeloma and malignant brain tumors. Furthermore, this axis is also correlated to the progression of chronic lymphocytic leukemia (CLL) and acute lymphoblastic leukemia (ALL), and acute myeloid leukemia (AML). In addition, rheumatoid arthritis (RA) is caused mainly by CD4^+^ memory T cell accumulation in the inflamed synovium. It was reported that CXCL12 concentration is extremely elevated in the synovium of RA patients, and that CXCR4 is highly expressed on the surface of memory T cells. Further, CXCL12 stimulates migration of the memory T cells and thereby inhibits T cell apoptosis. This indicates that the CXCR4-CXCL12 interaction plays an essential role in the accumulation of T cells in the RA synovium. Taken together, CXCR4 is an attractive therapeutic target for these diseases, and our recent research concerning the development of several CXCR4 antagonists including downsizing is discussed in this review article.

## Development of CXCR4 Antagonists as Selective Inhibitors of X4-HIV-1 Entry

**Figure 2:**
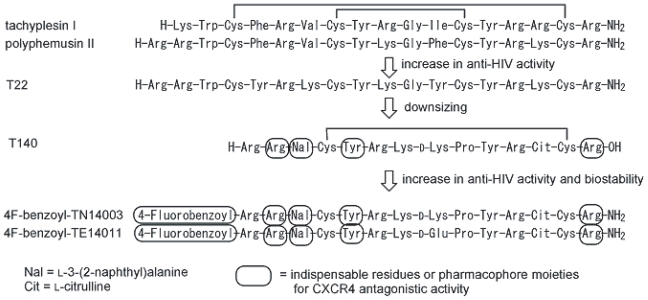
Structures of tachyplesin I, polyphemusin II, its analog T22, its downsized analog T140, its biostable analogs 4F-benzoyl-TN14003 and 4F-benzoyl-TE14011.

Self-defense peptides with antibacterial and antiviral activities, tachyplesins and polyphemusins, have been isolated from the hemocyte debris of the Japanese horseshoe crab (*Tachypleus tridentatus*) and the American horseshoe crab (*Limulus polyphemus*), which are 17-mer and 18-mer peptides, respectively (Fig. 2) (Nakamura et al. 1988; Miyata et al. 1989). Our preliminary structure-activity relationship studies of these peptides led to the development of T22 ([Tyr^5,12^, Lys^7^]-polyphemusin II) (Masuda et al. 1992; Nakashima et al. 1992) and its downsized 14-mer peptide, T140, which possess strong anti-HIV activity (Fig. 2) (Tamamura et al. 1998). T22 and T140 effectively block X4-HIV-1 entry into cells by binding specifically to CXCR4, and inhibit Ca^2+^ mobilization caused by CXCL12 stimulation against CXCR4 (Murakami et al. 1997; Xu et al. 1999; Murakami et al. 1999). In addition, a T140 analog exhibited a remarkable and significant delaying of the appearance of drug-resistant strains of HIV in passage experiments using cell cultures *in vitro* (Kanbara et al. 2001), and it was presumed that the T140 analogs would be useful for its suppressive effect against drug-resistant strains. Structural analysis revealed that T140 forms an antiparallel β-sheet structure supported by a disulfide bridge between Cys^4^ and Cys^13^, which is connected by a type II’ β-turn (Tamamura, Sugioka et al. 2001). Four amino acid residues that were contained in T140, Arg^2^, L-3-(2-naphthyl)alanine (Nal)^3^, Tyr^5^ and Arg^14^, were identified as residues indispensable for significant activity (Tamamura et al. 2000).

However, T140 is proven to be biologically unstable, and biodegradable in mouse/feline serum or in rat liver homogenate (Tamamura, Omagari et al. 2001; Tamamura, Hiramatsu, Kusano et al. 2003). When indispensable amino acid residues (Arg^14^ in serum; Arg^2^, Nal^3^ and Arg^14^ in liver homogenate) are deleted from the *N*- and the *C*-termini, the efficacy of degraded peptides is dramatically reduced. Modification of T140 analogs at both termini efficiently suppresses the above biodegradations and leads to development of novel and effective compounds that show highly CXCR4-antagonistic activity as well as increased biological stability. Further studies on the *N*-terminal modification found an electron-deficient aromatic ring such as a 4-fluorobenzoyl moiety at the *N*-terminus to constitute a novel pharmacophore for strong anti-HIV activity. The T140 analogs, which contain an *N*-terminal 4-fluorobenzoyl moiety, 4F-benzoyl-TN14003 and 4F-benzoyl-TE14011, have anti-HIV activity two orders of magnitude higher than that of T140 and enhanced biostability in serum/liver homogenates (Fig. 2) (Tamamura, Hiramatsu, Mizumoto et al. 2003).

## Cyclic Peptides with CXCR4 Antagonistic Activity Derived from T140

**Figure 3:**
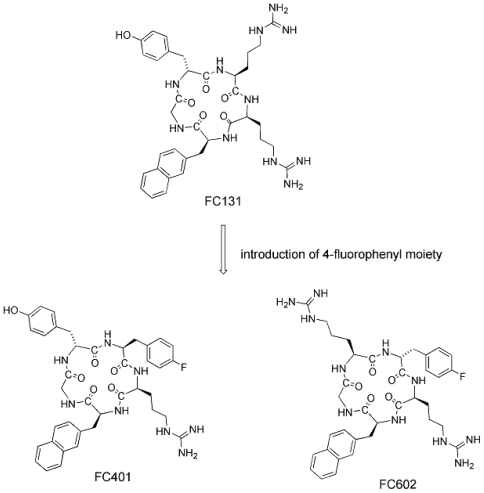
Structures of cyclic pentapetides FC131, FC401 and FC602.

Arg^2^, Nal^3^, Tyr^5^ and Arg^14^ of T140, which are located in close proximity to each other in space, are indispensable to high antagonistic activity against CXCR4 as described above. For downsizing of T140 analogs, a pharmacophore-guided approach was performed using cyclic pentapeptide libraries, which were composed of two L/D-Arg, L/D-Nal and L/D-Tyr in addition to Gly as a spacer. This approach led to FC131 [*cyclo*(-Arg^1^-Arg^2^-Nal^3^-Gly^4^-D-Tyr^5^-)], which showed strong CXCR4-antagonistic activity comparable to that of T140 (Fig. 3) (Fujii et al. 2003). Structural analysis of FC131 by NMR and simulated annealing molecular dynamics revealed the near-symmetrical pentagonal backbone structure.

A 4-fluorophenyl moiety found as a pharmacophoric moiety as described in the above section was introduced into cyclic pentapeptides. Since replacement of the phenol group of D-Tyr^5^ by a 4-fluorophenyl group did not cause the maintenance of high potency, the 4-fluorophenyl group was incorporated into position 1. The resulting compound, FC401 ([Phe(4-F)^1^]-FC131), shows significant CXCR4-binding activity (Fig. 3) (Tamamura et al. 2005). Next, since a second Arg residue is thought to be indispensable for high potency and an aromatic residue [L/D-Phe(4-F)] has been incorporated into position 1, four analogs [L/D-Phe(4-F)^1^, L/D-Arg^5^]-FC131 were synthesized based on replacement of D-Tyr^5^ by L/D-Arg^5^. Among these analogs, FC602, which is [D-Phe(4-F)^1^, Arg^5^]-FC131, shows the most potent activity, which is 10-fold greater than that of [D-Tyr^1^,Arg^5^]-FC131 (Fig. 3). Thus, FC602 is a novel lead, which involves a pharmacophore moiety different from the pharmacophore groups of FC131.

## A Linear Type of Low Molecular Weight CXCR4 Antagonists

**Figure 4:**
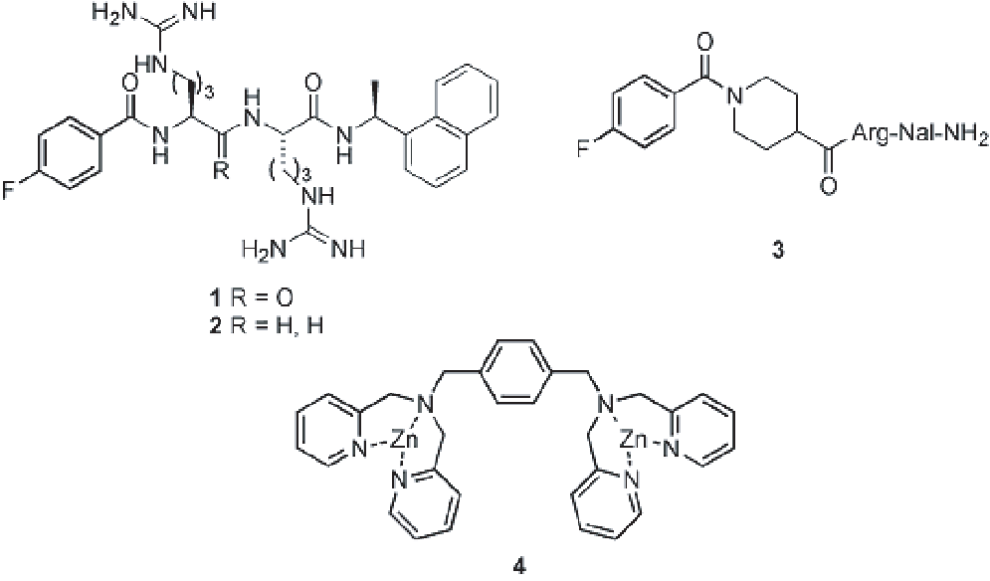
Structures of a linear type of low molecular weight CXCR4 antagonists.

Identification of a novel pharmacophore for CXCR4 antagonism, such as a 4-fluorobenzoyl or 4-fluorophenyl moiety, prompted us to develop a linear type of low molecular weight CXCR4 antagonists. By combining substructure units of the T140 pharmacophore and new pharmacophore moieties, several compounds were designed and synthesized using combinatorial chemistry. As a result, several linear compounds were found as moderate CXCR4 antagonists, such as compounds **1**–**3** shown in Figure 4 (Tamamura, Tsutsumi et al. 2006). These compounds are relatively weaker than a cyclic pentapeptide FC131. Thus, it is thought that conformational constriction based on a cyclic pentapeptide scaffold is critical for strong potency.

Anthracene derivatives possessing two sets of zinc(II)-2,2’-dipicolylamine complex were previously found as useful chemosensors that can selectively bind to phosphorylated peptide surfaces (Ojida et al. 2004). Several low molecular weight compounds bearing the complex structure were identified as selective CXCR4 antagonists (Tamamura, Ojida et al. 2006). Molecular superposition of structures of the zinc(II)-2,2’-dipicolylamine complex compound 4 and the cyclic pentapeptide FC131 was investigated as it provided the best fit with the maintenance of local energy minimizations of both of the structures (Fig. 4). The distance between two dipicolylamine moieties of compound 4 is estimated to be nearly equal to that between the two Arg side chains of FC131. Thus, the distance of these functional groups is thought to be essential for expression of CXCR4 antagonistic activity.

## Anti-Metastasis Activity of T140 Analogs Against Breast Cancer, Melanoma and Pancreatic Cancer

While CXCR4 and another chemokine receptor, CCR7, are highly expressed on the surface of human breast cancer cells, CXCL12 and a CCR7 ligand, CCL21, are highly expressed in lymph nodes, bone marrow, lung and liver, which form the common metastatic destinations of breast cancer. The CXCL12-CXCR4/CCR7-CCL12 axis might determine the metastatic destination of tumor cells and cause organ-preferential metastasis (Müller et al. 2001). Metastasis of breast cancer cells to the lung in mice was inhibited by neutralizing CXCR4 with anti-CXCR4 antibodies. We evaluated the inhibitory activity of our T140 analogs against the migration of breast cancer cells *in vitro* and metastasis of breast cancer cells *in vivo* (Tamamura, Hori et al. 2003). T140 analogs inhibited in dose-dependent manners the migration of a CXCR4-positive human breast carcinoma cell line MDA-MB-231 induced by CXCL12. Furthermore, the inhibitory effect of a bio-stable T140 analog, 4F-benzoyl-TN14003, was confirmed using experimental metastasis models of breast cancer, in which MDA-MB-231 cells were injected intravenously into the tail vein of SCID mice and trapped in the lung to which they migrated through the heart and the pulmonary artery. 4F-benzoyl-TN14003 was subcutaneously injected using an Alzet osmotic pump (DURECT Corp., Cupertino, CA, U.S.A.), and an effective suppression of tumor accumulation was then shown on the lung surface as a result of MDA-MB-231 metastasis. This suggests that small molecule CXCR4 antagonists, such as T140 analogs, can replace anti-CXCR4 antibodies as neutralizers of metastasis of breast cancer.

It was reported that an excessive expression of CXCR4 on B16 melanoma cells enhances the metastatic accumulation of the cells in mouse lung, and that a CXCR4 antagonist T22 blocks pulmonary metastasis in mice injected with CXCR4-transduced B16 cells (Murakami et al. 2002). We found that T140 analogs inhibited pulmonary metastasis in mice injected with B16 cells, which are not transduced with CXCR4 (Takenaga et al. 2004). Poly D,L-lactic acid (PLA) microcapsules containing a T140 analog, 4F-benzoyl-TE14011, was subcutaneously injected in experimental metastatic models of CXCR4-positive B16-BL6 melanoma cells. 4F-benzoyl-TE14011 is released in a controlled fashion from the PLA microcapsules for a long period *in vivo* with the maintenance of the level of the 4F-benzoyl-TE14011 concentration in blood. A single subcutaneous administration of 4F-benzoyl-TE14011-PLA significantly decreases the number of colonies ascribed to pulmonary metastasis of B16-BL6 cells. Thus, a controlled release of CXCR4 antagonists might lead to effective suppression of cancer metastasis.

While CXCL12 mRNA is expressed in pancreatic cancer tissues, CXCR4 mRNA is expressed both in pancreatic cancer tissues and in pancreatic cancer cell lines (AsPC-1, BxPC-3, CFPAC-1, HPAC and PANC-1) (Koshiba et al. 2000). CXCL12 stimulates induction of both migration and invasion of pancreatic cancer cells, AsPC-1, PANC-1 and SUIT-2, in dose-dependent manners *in vitro*. Thus, it suggests that the interaction between CXCL12 and CXCR4 is relevant to pancreatic cancer cell progression and metastasis. CXCL12-induced migration and invasion of these cells are suppressed by T140 analogs in dose-dependent manners (Mori et al. 2004). CXCL12 treatment of PANC-1 cells induces a drastic increase in actin polymerization (cytoskeleton), which causes the invasion and subsequent metastasis of malignant cells into tissues. T140 analogs effectively inhibit this phenomenon.

Furthermore, the CXCL12-CXCR4 axis is relevant to metastasis of SCLC and osteolysis in multiple myeloma. Thus, the blockade of this axis might be an effective remedy against these diseases (Hartmann et al. 2005; Zannettino et al. 2005).

CXCR4 is expressed in malignant cells in at least 23 different types of cancer (Balkwill 2004), including those discussed above. Antagonists of CXCR4 such as the T140 analogs might be useful lead compounds for the development of anti-metastatic agents in several types of cancer.

## Effect of T140 Analogs Against ALL and CLL

Growth and survival of ALL precursor B (pre-B) cells might be caused by mutual contact with bone marrow stromal layers through adhesive interactions between leukemia cells expressing CXCR4 along with integrins and stromal cells expressing CXCL12 and integrin ligands. Migration of these cells into stromal layers is stimulated by CXCL12, which is constitutively secreted at high levels by marrow stromal cells, since CXCR4 is highly expressed in the pre-B cells. T140 suppresses CXCL12-induced chemotaxis of the cells and attenuates their migration into bone marrow stromal layers. Furthermore, since T140 analogs enhance the cytotoxic and anti-proliferative effects of other anti-cancer agents such as vincristine and dexamethasone, T140 analogs might overcome cell adhesion-mediated drug resistance (CAM-DR) in ALL chemotherapy (Juarez et al. 2003).

On the other hand, B cell CLL, which is the most frequent leukemia in adults in Western countries, is caused by the accumulation of long-lived, monoclonal, malignant B cells in blood, secondary lymphoid organs and bone marrow. CLL B cells that highly express CXCR4 are activated by CXCL12 released from marrow stromal cells or nurselike cells. CXCL12-stimulation rescues the CLL B cells from apoptosis and contributes to their accumulation. Consequently, the CXCL12-CXCR4 axis should also be a therapeutic target of B cell CLL (Burger et al. 2000). Practically, T140 analogs inhibit chemotaxis of CLL B cells induced by CXCL12, their migration beneath marrow stromal cells and actin polymerization in dose-dependent manners, *in vitro* (Burger et al. 2005). Furthermore, T140 analogs attenuate the anti-apoptotic effect of CXCL12 and prevent stromal cells from protecting against spontaneous apoptosis of CLL B cells. Co-cultivation of CLL B cells with marrow stromal cells causes stromal CAM-DR, protecting CLL B cells from undergoing fludarabine-induced apoptosis. Treatment with T140 analogs re-sensitizes these B cells towards fludarabine-induced apoptosis T140 analogs might overcome CAM-DR which is a serious problem in the clinical CLL chemotherapy.

## Anti-RA Activity of T140 Analogs

The development of biological drugs such as monoclonal antibodies, which target inflammatory cytokines: tumor necrosis factor, TNF-α, interferon, IFN-γ, the interleukins, IL-1, IL-6, etc., has brought useful results in clinical RA therapy. However, complete curative effects have not yet been achieved. Thus, other drugs, which are not relevant to the functions of these cytokines, are required to improve RA chemotherapy. Since the CXCR4-CXCL12 axis plays a critical role in the accumulation of memory T cells in the RA synovium (Nanki et al. 2000), anti-RA activity of 4F-benzoyl-TN14003 was evaluated. Delayed-type hypersensitivity (DTH) reaction induced by sheep red blood cells (SRBC) was performed as an *in vivo* experimental model of the cellular immune response (Tamamura et al. 2004). Subcutaneous injection of 4F-benzoyl-TN14003 using an Alzet osmotic pump significantly suppressed the footpad swelling (the DTH response) in dose-dependent manners. Collagen-induced arthritis (CIA) in mice was adopted as the other *in vivo* experimental RA model. Several symptoms of arthritis: score increase, body weight loss, ankle swelling, limb weight gain, etc. were remarkably suppressed. Furthermore, the increase in levels of serum anti-bovine CII IgG2a antibody was apparently suppressed in mice treated with 4F-benzoyl-TN14003 subcutaneously using an Alzet osmotic pump after treatment with the bovine type II collagen (CII) emulsion booster. 4F-benzoyl-TN14003 exhibits an inhibitory effect towards the humoral immune response to CII. Thus, CXCR4 antagonists such as T140 analogs might also be useful leads for anti-RA agents.

## Other CXCR4 Antagonists

Several low molecular weight CXCR4 antagonists, which are not correlated to T140, have been reported to date (Mastrolorenzo, Scozzafava and Supuran, 2001; Scozzafava, Mastrolorenzo and Supuran, 2002). AMD3100 bearing two cyclam groups (Genzyme) (Schols et al. 1997), an *N*-pyridinylmethylene cyclam (monocyclam) AMD3465 (Genzyme) (De Clercq, 2002), AMD8665 without a cyclam group (Genzyme) (Seibert and Sakmar, 2004), AMD070 (Genzyme) (Vermeire et al. 2004), ALX40-4C (Ac-[D-Arg]_9_-NH_2_; NPS Allelix) (Doranz et al. 1997), CGP64222 (Cabrera et al. 2002), R3G (Cabrera et al. 2000), NeoR (Daelemans et al. 2000), a distamycin analog, NSC651016 (Howard et al. 1998) and a flavonoid compound, ampelopsin (Liu et al. 2004), have been identified as CXCR4 antagonists. Conjugates of AMD3100 and galactosylceramide (GalCer) analog have also been found as doubly-functionalized drugs (Daoudi et al. 2004). KRH-1636 (Kureha Chemical and Sankyo) is an orally bioavailable agent possessing *N*-pyridinylmethylene, Arg and naphthalene moieties (Ichiyama et al. 2003). A review of non-T140-related CXCR4 antagonists has been published elsewhere (Tamamura and Fujii, 2005).

## Conclusion

We have found strong anti-HIV agents, T22 and its downsized analog T140, identified as entry inhibitors that bind specifically to the chemokine receptor CXCR4 and thus inhibit entry of X4-HIV-1 to T-cells. T140 and its analogs also show effective activity against cancer metastasis, leukemia and rheumatoid arthritis. Cyclic pentapeptide FC131 was developed as a new low molecular weight CXCR4 antagonist by downsizing of T140. Furthermore, a linear type of low molecular weight CXCR4 antagonists involving aromatic compounds having the zinc(II)-2,2’-dipicolylamine complex structure have been found. These antagonists might be promising agents for clinical chemotherapy of multiple disorders such as HIV infection, cancer metastasis, leukemia and RA. However, blocking of the CXCL12-CXCR4 axis might be a risky procedure because CXCR4 plays a critical role in embryogenesis, homeostasis and inflammation in the fetus especially in the embryonic development of hemopoietic, cardiovascular and central nervous systems. Use of CXCR4 antagonists combined with CCR5 antagonists/fusion inhibitors might lead to a useful candidate for clinical AIDS chemotherapy.
